# The Effects of Osteopathic Manipulative Treatment on Brain Activity: A Scoping Review of MRI and EEG Studies

**DOI:** 10.3390/healthcare12131353

**Published:** 2024-07-06

**Authors:** Mirjam Bonanno, Giuseppe Alfredo Papa, Paola Ruffoni, Emanuele Catalioto, Rosaria De Luca, Maria Grazia Maggio, Rocco Salvatore Calabrò

**Affiliations:** 1 IRCCS Centro Neurolesi Bonino-Pulejo, Cda Casazza, SS 113, 98124 Messina, Italy; rosaria.deluca@irccsme.it (R.D.L.); mariagrazia.maggio@irccsme.it (M.G.M.); roccos.calabro@irccsme.it (R.S.C.); 2 Kinestudio, Via Albero Perroni, Terme Vigliatore, 97050 Messina, Italy; giuseppe.alfredo@gmail.com; 3 International College of Osteopathic Medicine, 20092 Milan, Italy; paola.ruffoni@assistenti.icomedicine.com; 4 Radiodiagnostic Unit, A.O. Papardo, 98158 Messina, Italy; emanuele.catalioto@gmail.com

**Keywords:** osteopathic manipulative treatment, brain activity, brain connectivity, fMRI, EEG, therapeutic touch

## Abstract

Osteopathic manipulative treatment (OMT) is a hands-on therapy aiming to achieve the global homeostasis of the patient. OMT focuses on treating the somatic dysfunctions characterized by tissue modifications, body asymmetry, and range-of-motion restrictions. The benefits related to OMT are thought to be associated with the interconnectedness of the body’s systems and the inherent capacity for self-healing. However, whether OMT can influence brain activity, and, consequently, neurophysiological responses is an open research question. Our research investigates the literature to identify the effects of OMT on brain activity. The main purpose of the research question is: can OMT influence brain activity and consequently neurophysiological responses? A scoping review was conducted, searching the following databases: PubMed, Google Scholar, and OSTEOMED.DR (Osteopathic Medical Digital Repository), Scopus, Web of Science (WoS), and Science Direct. The initial search returned 114 articles, and after removing duplicates, 69 were considered eligible to be included in the final sample. In the end, eight studies (six randomized controlled trials, one pilot study, and one cross-over study) were finally included and analyzed in this review. In conclusion, OMT seems to have a role in influencing functional changes in brain activity in healthy individuals and even more in patients with chronic musculoskeletal pain. However, further RCT studies are needed to confirm these findings. Registration protocol: CRD42024525390.

## 1. Introduction

Osteopathic manipulative treatment (OMT) is a hands-on therapy aimed at the achievement of global homeostasis of the subject [1]. OMT focuses on the person-centered approach, treating the somatic dysfunctions (SDs) characterized by tissue modifications, body asymmetry, and motion restrictions [2]. An SD can be defined as an impaired regulatory function with palpable inflammatory signs in different body regions, potentially distant from the pain location. Some authors suggested that the clinical application of the osteopathic five models (biomechanical, respiratory/circulatory, neurological, biopsychosocial, and bioenergetic) could provide a framework for the individuation and the diagnosis of an SD [1,3]. In addition, the palpatory findings and the clinical signs of SD should be interpreted in a step-by-step decision-making process, which includes the case history, injury, chronicity, and evidence of sensitization [1,4]. However, how osteopaths manage the decision-making process and why they choose one type of treatment over another is still unclear. In general, OMT is considered a whole-body patient-centered intervention, primarily focused on sustaining a person’s health [5]. In this sense, OMT promotes a holistic person-centered approach, interacting with the patient through direct and indirect manual techniques [2,6]. The benefits related to OMT are thought to be associated with the interconnectedness of the body systems and the inherent capacity for self-healing. From a biological point of view, OMT can influence the release of endocannabinoids, including serotonin, arginine, vasopressin, cortisol, and oxytocin [7,8]. Consequently, oxytocin release modulates the activity of the anterior insular cortex (AIC), which is strictly involved in response to noxious stimuli, thus reducing physical relief [7,9]. Several authors suggest that therapeutic touch has a potential anti-inflammatory effect, due to the reduced activity of the hypothalamus-pituitary-adrenal (HPA) axis [7,10]. According to Rechberger and colleagues [11], osteopathic techniques in the cranial field (i.e., compression of the fourth ventricle (CV4), suboccipital myofascial release) can act on the autonomic nervous system (ANS), inducing positive changes, and influencing the sympathovagal balance. However, whether OMT can influence brain activity and consequently neurophysiological responses is an open research question [6]. Research studies have demonstrated that other manual therapies, such as chiropractic manipulation, spinal mobilization, and therapeutic touch have an immediate effect on brain activity [12,13]. For example, Isenburg and colleagues [13] demonstrated that joint mobilization and spinal manipulation, based on Maitland’s method, can reduce low back pain (LBP). Furthermore, these authors showed that this pain reduction occurred via the modulation of salience network connectivity to sensorimotor, affective, and cognitive processing regions. 

Brain activity can be studied in different and non-invasive ways, including magnetic resonance imaging (MRI) and image processing methods, such as diffusion tractography, in order to quantify structural connectivity. Structural brain connectivity consists of the anatomical organization of the brain through fiber tracts [14,15]. On the other hand, functional brain connectivity refers to statistical dependence between time series of electro-physiological activity and (de)oxygenated blood levels in distinct brain regions. Electrophysiological brain activity can be easily recorded with electroencephalography (EEG) [16]. These methods record changes in averaged post-synaptic potentials, providing an idea of neural functioning [17]. Otherwise, the blood-oxygen-level-dependent (BOLD) technique, obtained through functional magnetic resonance imaging (fMRI), has shown a great ability to highlight active brain areas [18]. The BOLD signal reflects changes in deoxyhemoglobin guided by specific changes in brain blood flow and blood oxygenation [15]. These changes reflect underlying neuronal activity through a process known as neurovascular coupling, offering an indirect insight into neural functioning [18]. To this end, the detection of structural and functional changes in the brain reflects the neurophysiological functioning of the nervous system [19]. 

This scoping review aims to provide a comprehensive insight into relevant studies in the literature about the effects of OMT on brain activity, measured with MRI and EEG instrumentation, shedding some light on this under-debate field of research. 

## 2. Materials and Methods

### 2.1. Research Question 

Our research investigated the literature to identify the effects of OMT on brain activity measured with MRI and EEG. According to the acronym PCC (Population/Problem, Concept, Context), our research focused on the main question: “Can OMT influence brain activity and consequently neurophysiological responses? And how?”. We considered studies on healthy adults, and/or people suffering from musculoskeletal chronic pain, as the population. The concept refers to the application of OMT or osteopathic techniques and how they can influence brain activity measured with MRI and EEG in the context of a manual therapy setting. The protocol of this scoping review was registered on PROSPERO with the registration number: CRD42024525390, following the Preferred Reporting Items for Systematic and Meta-analyses extension for Scoping Review (PRISMA-ScR). The choice of conducting a scoping review was determined by the research topic. A scoping review provides a summary and explanation of key concepts and research gaps, and it is thought to be a precursor of a systematic review [20]. 

### 2.2. Eligibility Criteria 

This review included primary research articles, such as randomized controlled trials (RCTs), cross-over studies, and pilot studies, about OMT reporting effects on brain activity measured with MRI and EEG in healthy adults and individuals with non-specific musculoskeletal pain (e.g., low back pain, headache, non-specific cervical pain), compared to placebo or sham treatments. Articles were included if they presented the following criteria: (i) RCTs, pilot studies, cross-over study methodologies; (ii) published in peer-reviewed journals; (iii) written in English because the majority of high-impact and widely cited studies are available in this language; (iv) published between 2013 and March 2023 to ensure up-to-date data. Specifically, articles were excluded if, as follows: (i) the focus was on other types of complementary treatments (i.e., acupuncture, Chinese medicine); (ii) unspecific manual treatments (e.g., chiropractic techniques, general manual therapy, and other manual treatments like the Maitland method). The choice of this reduced inclusiveness is linked to the need to understand whether OMT modulates brain activity in both healthy and chronic pain conditions; (iii) animal models; (iv) grey literature, including sources reports, theses, conference papers, and non-peer-reviewed articles, because these sources may not ensure the same level of quality and reliability as studies published in the academic literature; and (v) theoretical models, methodological approaches, algorithms, and basic technical descriptions; animal studies; conference proceedings, or reviews. 

### 2.3. Information Sources and Search Strategy 

To guarantee a comprehensive and up-to-date search, the following databases were consulted: PubMed, Google Scholar, and OSTEOMED.DR (Osteopathic Medical Digital Repository), Scopus, Science Direct, and Web of Science (WoS). These databases were selected as they cover the majority of peer-reviewed, allied studies in the health literature. Two fundamental keywords were searched: osteopathic manipulative treatment and brain connectivity. In particular, the search strategy used is reported in Table 1 for each database source. The searches were limited to the title and abstract in this phase. Moreover, the references of the selected articles were also analyzed to obtain a complete search. The articles were evaluated according to their title, abstract, and text, and selected based on their scientific validity, as per the author’s assessment.

### 2.4. Selection Process, Data Collection and Data Items

Therefore, articles were retrieved and screened, according to titles and abstracts, following the above inclusion criteria using a web application, namely, RYAAN [21]. After this preliminary phase, the included articles were screened according to the main text for the data charting process. The list of articles was then refined for relevance, revised, and summarized, with the key themes identified from the summary based on the inclusion/exclusion criteria. The following information was considered: authors, year, and type of publication (e.g., RCTs, clinical trials, pilot studies, cross-over studies), characteristics of the participants involved in the study, type of intervention used, brain activity, instrumented evaluation, and major findings.

## 3. Results

**Figure 1:**
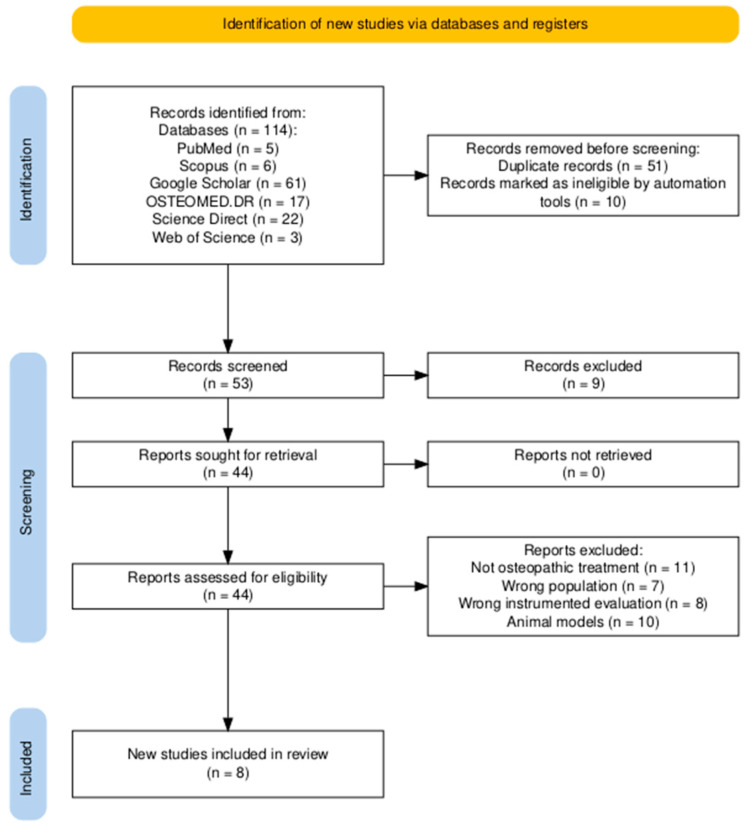
The Preferred Reporting Items for Systematic reviews and Meta-Analyses (PRISMA) flowchart for the inclusion of the studies [22].

The initial search returned 114 articles, and after removing duplicates, 53 articles were considered eligible for inclusion in the final sample. During the screening process, articles describing theoretical models, methodological approaches, algorithms, and basic technical descriptions (n = 10); animal studies (n = 10), conference proceedings (n = 4), or reviews (n = 3) were excluded. Then, 36 papers were removed due to wrongly instrumented evaluations or treatments not specifically based on osteopathic principles. After thoroughly reviewing the manuscripts, eight articles were included in the final sample (Figure 1).

### 3.1. OMT Intervention

From our findings, it emerges that authors administered different OMT interventions. In detail, some authors [23,24,25] performed a specific technique, like CV4 [23,24], sacral techniques [23], and spinal manipulation and/or therapeutic touch [25]. In these studies, osteopathic techniques were described, and were similar to each other, except for Gay and colleagues [25], who performed a different treatment than Miana et al. [24] and Cella and colleagues [23]. 

Otherwise, the majority of the authors [26,27,28,29,30] administered an OMT based on the specific SDs found during the physical examination. The administered osteopathic techniques included articular and myofascial techniques, balanced ligamentous tension, visceral manipulations, and osteopathy in the cranial field, according to the SDs found in the body region. Specifically, Tamburella and colleagues [26] as well as Tramontano and colleagues [27] mostly performed visceral manipulations, cranial treatment, and myofascial release in asymptomatic individuals with SDs mainly on the cervical, head, and abdomen regions. Therefore, these authors did not define a specific protocol of intervention and did not solely investigate one specific technique. However, some authors [26,27] performed mostly visceral manipulations and cranial-sacral techniques, focusing primarily on the head, and abdomen regions. Cerritelli and co-authors [28,30] administered indirect techniques, using light and gentle touch to correct the SD by applying the Sutherland’s point of maximum freedom (balance point) model. 

All the included studies compared the experimental intervention (OMT) with a placebo or sham treatment. Specifically, Tramontano and colleagues [27], as well as Tamburella and co-authors [26], performed a similar placebo treatment lasting 25 min. In both studies, the osteopath stood next to the bed with the patients lying prone and then supine. The body locations of touch were the lumbar and dorsal spine, shoulders, hips, upper and lower limbs [27], neck, sternum, and chest. In addition, only Tramontano and colleagues [27] and Tamburella and colleagues [26] administered a de-blinding questionnaire about the perceptions of the subjects on the treatments received.

Cerritelli and others [28,30] administered an osteopathic-like manual assessment to the control group without focusing on areas with SDs. The body parts identified in the protocol were the lumbar spine, sacrum, pelvis, diaphragm, upper thorax, cervical spine, and cranium. They then applied a static or dynamic gentle touch on a predefined number of anatomical areas. Miana and co-authors [24] administered a sham CV4 technique, in which the operator simulated the real CV4 procedure without applying any tactile pressure on the occiput between the occipitomastoid sutures. Once placement was achieved, the operator’s hands remained motionless for 10 min. Similarly, Cella and others [23] conducted the sham CV4 and sacral technique with their hands in the same position as the corresponding active techniques, applying a non-therapeutic touch. Additionally, Cerritelli and colleagues [29] divided participants into the following two groups based on the operator’s attention: (1) the tactile perception from his hands (OTA group), or (2) a repeated auditory stimulus (OAA group). In the OTA group, the operator focused on tactile sensations such as the consistency, density, temperature, responsiveness, and motility of the tissue (e.g., myofascial movements). In contrast, the operator in the OAA group directed his attention to acoustic stimuli (beeps) delivered through headphones. Differing from the others, Gay and colleagues [25] divided patients into the following three manual-based intervention groups: spinal manipulation; spinal mobilization; and therapeutic touch (see Table 2 for details).

Regarding the duration of the sessions, Tramontano and colleagues [27], as well as Tamburella and co-authors [26] administered 45 min of treatment for both the OMT and placebo groups. Cerritelli and colleagues (2020 and 2021) conducted four weekly sessions of OMT or placebo, each lasting 30 min. Conversely, Miana and co-authors [24] administered 10 min of the CV4 technique and a sham technique once a week. Notably, Cella and co-authors [23] employed a crossover design with two experimental sessions, each lasting approximately 50 min, separated by a 4 h washout period. 

### 3.2. Instrumented Evaluation: MRI and EEG 

From the included studies, six articles [23,24,25,26,27,29] studied brain activity after OMT in healthy adults, and the remaining two [28,30] considered patients with chronic non-specific LBP. The majority (six out of eight) of the studies [25,26,27,28,29,30] investigated brain activity and connectivity by using 3T-MRI, and in four articles [27,28,29,30] fMRI and BOLD techniques were also performed. Tamburella and co-authors [26] were the only authors to test morphologically and functionally cerebral blood-flow (CBF) in MRI by using the pseudo-continuous arterial spin (pcASL) technique. Indeed, heterogeneity among MRI protocols was found. For example, Cerritelli and colleagues [28,30], and Tramontano and colleagues [27] used different MRI parameters (e.g., voxel measures, matrix, and slice thickness) to study brain activity, as reported in Table 2. 

Otherwise, Miana and others [24] and Cella and colleagues [23] evaluated neurophysiological brain activity by using 20-channel EEG. In detail, Cella and others [23] derived EEG signals from the occipital O1 and O2 electrodes, and the electrode impedance was kept below 10 kΩ. In addition, these authors continuously recorded EEG signals during each technique application and each resting state, with a 250 Hz sampling rate. Unlike Cella and others [23], Miana and colleagues [24] registered EEG signals before and after the administered osteopathic techniques. In addition, the EEG was performed in a dedicated space to avoid noises. 

### 3.3. Brain Activity Findings

**Figure 2:**
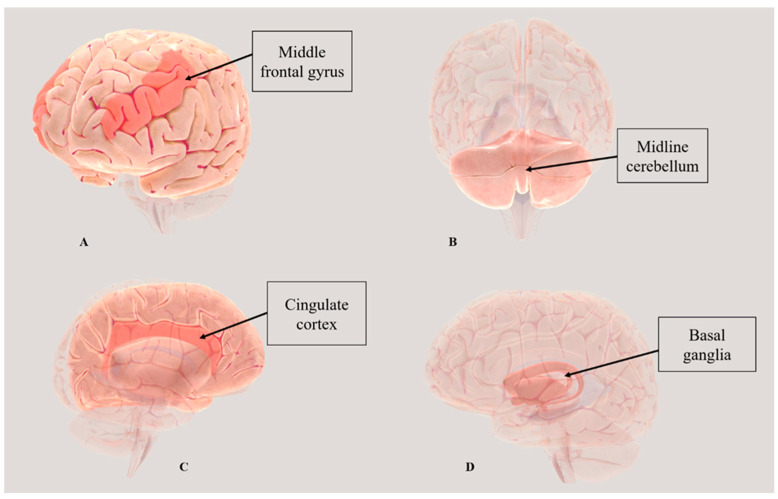
Reports on the most prevalent brain areas activated after OMT according to the included studies. Created using BrainFacts. Legend: (**A**) middle frontal gyrus; (**B**) midline cerebellum; (**C**) cingulate cortex; and (**D**) basal ganglia. Supporting evidence: (**A**) [27,30]; (**B**) [27]; (**C**) [26,27,28,30]; and (**D**) [27].

Despite the discrepancies related to MRI protocols, the pattern of brain activity showed some similarities among the authors. It seemed evident that brain regions involved in processing and modulating pain were reported to be more activated after the treatment (see Figure 2). In particular, Cerritelli and co-authors [30] found that brain analysis performed after OMT in individuals suffering from chronic LBP showed statistical differences. The authors found that OMT sessions caused a significant CBF decrease in brain areas related to pain, such as the left posterior insula, left ACC, and left thalamus, and consequently in heart rate variability. Before OMT, patients affected by chronic LBP showed increased baseline rCBF in pain-related brain regions such as the left posterior insula, left anterior cingulate cortex, and left thalamus. After OMT, there was a decrease in rCBF in these regions, along with an increase in rCBF in areas associated with pain modulation, including the left posterior cingulate cortex, bilateral striatum, and right anterior insula. These changes were correlated with self-reported pain scores, indicating the impact of OMT on neurophysiological correlates related to pain perception and modulation.

Interestingly, Cerritelli and others [29] demonstrated that operator attention sustained over time can elicit functional connectivity in participants who are receiving manual treatment. The authors revealed an increase in anticorrelation between PCC and the right insular cortex as well as the inferior frontal gyrus, in participants who received OMT. 

Surprisingly, Tramontano and colleagues [27] reported the activation of the midline cerebellum, basal ganglia, motor pathways, and the emotional/autonomic network, with specific activation of the amygdala, after the OMT intervention.

Otherwise, only two authors [23,24] used 20-channel EEG to detect changes in brain activity after osteopathic intervention. Both studies [23,24] found improvements in the activation of the alpha band after the CV4 technique.

## 4. Discussion

As far as we know, this is the first attempt to review studies in the literature about the effects of OMT on brain activity. Dal Farra and colleagues [31] have mapped the literature on the biological effects produced by OMT, including neurophysiological ones. Notably, they observed that while the studies demonstrated cardio-circulatory and respiratory effects related to OMT, these findings were still linked to changes in the autonomic nervous system (ANS). Therefore, although OMT can be applied to various anatomical areas for different clinical conditions, the nervous system may be the primary target of osteopathic treatment. Consequently, the osteopathic manual inspection could be an effective tool for assessing nervous system function, as hypothesized by the authors [31]. 

In our review, we specifically focused on brain activity measured with MRI and EEG. Some of the included articles [26,27,28,29,30] showed that after the OMT intervention, a change in cerebral perfusion and the activation of brain connectivity occurred, especially in those brain areas that are also involved in the “pain matrix”. The pain matrix, or neuromatrix theory, was conceptualized by Melzack [32], who stated that when noxious stimuli are modulated, a complex pattern in brain areas is activated. Interestingly, changes in brain activity, detected with MRI, have been found in patients with chronic LBP and in healthy individuals with SDs [27,28,30]. In this sense, Ponzo and colleagues [33] have shown that SDs may be associated with altered afferent input to the CNS, causing plastic neuronal changes. In addition, there was a difference between the two studies by Cerritelli and co-authors [28,30] and the other six studies [23,24,25,26,27,29] performed in healthy individuals. Specifically, patients with chronic LBP showed, before the OMT intervention, significant activation of the bilateral insula, bilateral cingulate cortex, bilateral sensory-motor cortex, and medial prefrontal cortex, which was not present in healthy individuals. This aspect may be related to the prolonged nociceptive stimuli caused by injury or inflammation that contribute to long-term alterations and plastic changes in the CNS [34]. Despite these differences, a common finding among the results of the included studies was the increased CBF in specific brain areas after the OMT, such as the insula, amygdala, motor cortex, thalamus, and cingulate cortex, as shown in Figure 2. 

Regarding thalamus activation, Mohamadi and others [35] investigated the role of the positional release technique in inducing changes in central sensitization in people with chronic headache tension. These authors found that the positional release technique in the neck muscles (e.g., trapezius, sternocleidomastoid, rectus capitis, multifidus, and others) was effective in reducing symptoms related to headache without inducing changes in metabolic brain profile related to central sensitization. The only metabolic change was at the thalamus level, which is the first destination center in the brain for sensory inputs. In this sense, the increased activation of the thalamus and related metabolite changes are related to the increased sensory inputs through positional release. In line with these findings, Isenburg and co-authors [13] found that manual therapy at the lumbar spinal level, based on Maitland’s method, was effective in reducing immediate pain symptoms in participants with chronic LBP. Interestingly, these authors demonstrated that manual therapy was associated with an increased salience network connectivity, a change not observed in the control group, which received only mobilization. Therefore, this may be explained by the somatosensory component of manual therapy, which alters the top-down processing of pain immediately following the manipulation. This occurs through increased salience network connectivity to the primary motor cortex [13]. In addition, the salience network connectivity was related to ventral lateral posterior, medial dorsal, and ventral posterior lateral nuclei of the thalamus [36]. The medial dorsal nucleus within the thalamus is implicated in both limbic and/or emotional processing as well as in cognitive functions, such as memory, through its interactions with the PFC [13]. In line with these findings, Tramontano and colleagues [27] showed that, after OMT, specific changes occurred in healthy adults, not only in the nociceptive/emotional/autonomic network but also in the sensorimotor and postural network, involving the midline cerebellum, basal ganglia, and prefrontal cortex. Therefore, the activation of the ventral lateral posterior nucleus was also found by Cerritelli and colleagues [30]. This aspect could suggest that this thalamus nucleus has a role during passive joint movement (e.g., manual therapy) and acts as an intermediary hub connecting the cerebellum and motor cortex. Consequently, manual therapy, as well as OMT, may impact various facets of pain processing, encompassing somatosensory, affective, and cognitive or memory domains, by regulating salience processing and allocating attentional resources via the connectivity of salience network to the thalamus nuclei [13]. 

Furthermore, chronic musculoskeletal pain, like LBP, can be associated with maladaptive neuroplastic changes in the grey matter, particularly in the cortical-limbic structures and emotional cerebral pathways that are involved in processing pain [34]. According to some authors, chronic pain stimuli can cause a decrease in grey matter density in the insular cortex, primary somatosensory cortex, motor cortex, hippocampus, amygdala, and nucleus accumbens [37]. It is noteworthy that these brain regions are involved also in processing cognitive and emotional information. In addition, these maladaptive changes in plasticity may also occur in conduction pathways from the peripheral nervous system to the CNS, contributing to the onset, progression, and persistence of chronic pain [34,37]. In this vein, the administration of osteopathic techniques, such as balanced-ligamentous, balanced-membranous, and fluidic techniques, was found to be effective in modulating the activation of the pain matrix, as demonstrated by Cerritelli and colleagues [28,30].

Moreover, several authors [25,26,28,29] reported the activation of PCC after OMT. Specifically, PCC is a critical node of the central autonomic network, which controls preganglionic sympathetic and parasympathetic motoneurons, having an important involvement in the parasympathetic functioning control [38]. These findings could suggest a possible modulation of the autonomic system provided by OMT, as confirmed by a previous systematic review [11]. According to Tamburella and co-authors [26] the whole-body OMT intervention, and not only cranial techniques, can locally modify the vascular activity, acting on the ANS. However, the interaction between brain and vascular activity and its involvement pre- and post-OMT were not investigated in all the included articles. Only two authors [26,30] have considered this; therefore, it should be further investigated in future studies to understand the role of OMT in influencing ANS, and the “brain–heart” axis. Additionally, the activation of the amygdala after the treatment, in the included papers, could suggest a rewarding role provided by OMT, increasing participants’ motivation [39].

Another critical point that should be discussed is the heterogeneity of the osteopathic techniques used by the authors. To this aim, some authors oriented the OMT to the treatment of SDs detected during the objective postural examination [26,27,28,29,30] while other authors used solely specific cranial techniques like CV4 [23,24]. It becomes evident that neurophysiological responses in this context can be highly variable and difficult to replicate [31]. Therefore, the positive results observed in the included studies should be considered as preliminary indications rather than definitive evidence of the brain activity changes induced by OMT. However, it should not be forgotten that what distinguishes osteopathy from other manual approaches is the person-centered vision. A distinctive aspect of osteopathy is its conceptualization of human health as dependent on the proper integration and functioning of various systems. Osteopaths identify body dysfunctions by assessing changes in movement patterns, tissue textures, and tenderness [31]. Consequently, SD is viewed as an impaired regulatory function, often associated with local inflammatory signs that can be evaluated through palpatory skills in the body [3,4]. Therefore, investigating the neurophysiological plausibility of OMT requires studying a range of biological changes that result from osteopathic approaches [31]. 

Furthermore, both Miana and others [24], and Cella and others [23], found an increase in alpha power after the CV4 technique, thus suggesting a condition of more internal attention and active inhibition of external sensory input. In line with these results, Cerritelli and colleagues [28,30], supported the effect of OMT in influencing the activation of specific brain interoception-related areas in patients with LBP. Given this, the question remains open as to whether it is global OMT that improves brain activity, or rather, specific cranial osteopathic treatment. 

Furthermore, all studies included young participants (around 20–30 years) and adults (around 40–42 years) people. This aspect should be considered since brain connectivity is sensible to change in the first 40 s, as demonstrated by some authors [40,41]. According to Damoiseaux [42], older adults can show decreased within-network connectivity, which appears to be related to cognitive decline. However, the role of OMT in inducing a modification of brain activity in older adults remains an open question, since no study has investigated this important issue yet.

After the 40s, the brain starts to undergo a radical “rewiring”, which has tangible effects on cognition, including interoception. In particular, “interoception” refers to the ability to perceive inner body sensations, such as heartbeat, respiration, and other visceral cues, influencing overall well-being. Alfano and co-authors [43] found significant correlations between interoception and brain connectivity in the salience network and fronto-temporal–parietal brain areas. In this sense, osteopathic manipulations could stimulate afferent neural pathways, even those involved in interoception, fostering the mind–body link. Specifically, Cerritelli and colleagues [29] found a negative correlation between PCC and the right insular cortex, suggesting that it could be a re-representation of interoceptive information on the right insular cortex. The insula is thought to sustain the early convergence of sensory and affective signals about the body, contributing to so-called “interoceptive processing”. In this sense, the recently discovered “CT-fibers” have been shown to project to insular brain regions, characterizing these afferents as an interoceptive modality and giving information about the affective, and physiological, state of the body, increasing body awareness [44]. However, research specifically linking OMT to interoception is limited, and its role in influencing neural plasticity should be also clarified. 

Interoception, through its impact on sensory inputs, individual differences, learning and memory, brain damage recovery, and experience-dependent plasticity, could influence neuroplasticity [41]. This is why future and larger studies should investigate whether OMT can stimulate those neural circuits involved in interoception, and if it can influence neuroplasticity. It could be a big step forward for research, as OMT could also be applied in clinical practice in those conditions where there is a brain injury, to promote functional recovery. 

On the other hand, the “toucher” is not solely a mere executor of the manual technique but is actively involved. Some studies demonstrated that stroking another’s skin is perceived as pleasant by the toucher, and it is associated with more positive, sensory experiences when targeted to activate the CT system [45], like during OMT. These findings suggest that the human brain is inherently wired not only to receive but also to deliver touch. In line with these results, Cerritelli and colleagues [29] highlighted the impact of attention states on brain functional connectivity during touch, emphasizing the role of operator attention in modulating neural responses. Delivering touch in hands-on care is crucial to communicating one’s cognitions and perceptions, facilitating the “mental state alignment” between osteopath and patient. In this way, the osteopath, through touch, aims to modify the patient’s symptoms, promoting allostatic regulation, and thus consolidating interpersonal relationships [7,46]. It may be this latter aspect that distinguishes experimental treatment (e.g., OMT) from placebo or sham treatment. In fact, most of the articles included in this review reported a comparison with placebo or sham treatment, consisting of unspecific and/or gentle touch, without therapeutic intention. It is noteworthy that the operator should be trained in performing sham technique [47]. In fact, the operator should pay attention to avoid any specificity in sham treatments [47]. In addition, several authors [26,27,28,30] considered some pre-defined anatomical areas, without the identification of SD, on which to apply the placebo or sham treatment. According to Cerritelli and co-authors [48], sham treatment should resemble active treatment in every aspect except the technique used. Applying the placebo–drug paradigm, one could argue that the true effect of the “active ingredient” in OMT is equivalent to the effects of real OMT minus the effects of placebo/sham OMT [48]. This implies that the technique should be the only differentiating factor between real and placebo OMT [48]. 

The main limitation of this scoping review that needs to be addressed is the lack of assessments of quality and the risk of bias in the selected papers. However, the main purpose of this review was to examine the available literature on the effects of OMT on brain activity measured with MRI and EEG. A systematic review should be considered shortly to better highlight the validity of the studies and the effectiveness of the OMT in influencing brain activity and, consequently, neurophysiological responses, as the literature on this topic is still insufficient. In addition, a quality assessment of the studies could be performed (e.g., using the PEDro scale [49] or RoB tool [50]) to provide the reader with clearer information on the quality of the selected manuscripts.

Furthermore, the selected studies have some limitations, such as small sample sizes, a lack of long-term follow-up evaluations, heterogeneity related to OMT, placebo and sham treatments, and the MRI protocols performed. This latter point could have also affected the studies’ results, as some MRI protocols allowed a more detailed view of brain connectivity through the use of a reduced FOV. In this sense, future studies should be homogenous in terms of MRI protocols and placebo and sham treatments, to make the results reproducible. 

## 5. Conclusions

In conclusion, OMT seems to have a role in promoting functional changes in brain activity in healthy adults and participants with chronic LBP. From our literature analysis, it seems that there is not a specific technique able to modulate brain activity; instead, the global osteopathic approach appears to be better. However, further RCT studies are needed to confirm these findings. In addition, future studies should investigate the role of OMT in influencing brain connectivity and neurophysiological responses. Future research should also compare healthy controls with SDs with patients affected by chronic musculoskeletal pain, considering clinical outcomes like quality of life and psychological well-being.

## Figures and Tables

**Table 1:** Search strategy used for the selection of the studies.

Database	Search Query	Search Fields	Filters
PubMed	((“osteopathic manipulative treatment”) OR (“manual therapy”)) AND ((“brain connectivity”) OR (“fmri”))	All fields	Date from 2013 to 2023
Google Scholar	“osteopathic manipulative treatment” OR “manual therapy” AND “brain connectivity”	NA	Date from 2013 to 2023 Study type: scientific articles
OSTEOMED.DR	osteopathic manipulative treatment OR manual therapy AND brain connectivity	All fields	Date from 2013 to 2023
Scopus	osteopathic AND manipulative AND treatment AND brain AND connectivity	Article title, Abstract, Keywords	Date from 2013 to 2023
Science Direct	“osteopathic manipulative treatment” or “manual therapy” and brain connectivity	Article title, Abstract, Keywords	Date from 2013 to 2023
Web of Science	osteopathic manipulative treatment AND brain connectivity	All fields	Date from 2013 to 2023

**Table 2:** Description of the studies included.

Reference	Study Design	Study Sample *	Intervention	Instrumented Evaluation **	Major Findings
(Tamburella et al., 2019) [26]	RCT single blinded	30 asymptomatic healthy adults EG: 15; age: 28.0 ± 5.5; CG: 15, age: 28.0 ± 5.5;	EG: OMT: - Articular and myofascial techniques; - Balanced ligamentous tension; - Visceral manipulations; - Cranial-sacral techniques). CG: passive touch	- 3T MRI (Philips Achieva): acquiring sixty tag-control image pairs with 29 axial slices (3 × 3 × 4.5 mm voxel resolution, matrix 80 × 80, and a 0.5 mm inter-slice gap). - Whole-brain CBF using pcASL (TR = 4000 ms, TE = 10 ms, label duration of 1650 ms, and a post-label delay of 1525 ms). - High-resolution T1-weighted whole-brain structural image was recorded with 1 × 1 × 1 mm voxels.	OMT produced significantly effects on: - PCC perfusion; - increase in CBF as a short-term effect; - These opposite effects may be rely on parasympathetic/sympathetic modulation
(Tramontano et al., 2020) [27]	RCT single blinded	24 asymptomatic healthy adults with SDs EG: 12; age: 28.0 ± 5.5; CG: 12, age: 25.4 ± 3.2;	EG: OMT: - Articular and myofascial techniques; - Balanced ligamentous tension; - Visceral manipulations; - Cranial-sacral techniques). CG: passive touch	- 3T MRI (Philips Achieva) using a (T2*)-weighted imaging sequence sensitive to BOLD signals: TR = 3 s, TE = 30 ms, matrix size of 80 × 80, FOV = 224 × 224, slice thickness of 3 mm, flip angle of 90 degrees, and acquisition of 50 slices across 240 volumes. - High-resolution T1-weighted whole-brain structural scan was conducted with 1 mm × 1 mm × 1 mm voxels.	OMT produced a specific functional and reversible connectivity re-arrangement of: - Premotor cortex, basal ganglia, and midline cerebellum; - Emotional/autonomic network, with specific activation of the amygdala.
(Cerritelli et al., 2020) [28]	RCT	29 right-handed patients with CLBP EG: 15, age: 41.8 ± 6.6; CG: 14, age: 42.7 ± 8.0;	EG: four sessions, per week, of OMT, lasting 30 min each, consisting of: - balanced-ligamentous tension; - balanced membranous; - fluidic techniques; CG: sham treatment (no application of any type of osteopathic technique or procedure).	- 3T MRI (Philips Achieva): matrix 256 × 256, FOV = 256 mm, slice thickness = 1 mm, no gap, in-plane voxel size = 1 × 1 mm, flip angle = 12°, TR = 9.7 ms, and TE = 4 ms; - BOLD fMRI data using a gradient-echo T2*-weighted echo-planar imaging sequence: matrix 80 × 80, voxel size 3 mm × 3 mm × 3.5 mm, SENSE 1.8, TE = 30 ms, TR = 1.8 s, with 185 volumes per run.	OMT elicited a specific BOLD activity in targeted areas related to interoception. OMT produced effects on - rINS, lINS; - ACC; - left striatum; - rMFG.
(Cerritelli et al., 2021) [30]	RCT	29 right-handed patients with CLBP EG: 15, 41.8 ± 6.6; CG: 14, 42.7 ± 8.0;	EG: four sessions, per week, of OMT, lasting 30 min each; CG: sham treatment.	- 3T MRI (Philips Achieva): matrix 256 × 256, FOV = 256 mm, slice thickness = 1 mm, no gap, in-plane voxel size = 1 × 1 mm, flip angle = 12°, TR = 9.7 ms, and TE = 4 ms. - BOLD fMRI data were collected; - Perfusion imaging with a pcASL sequence.	OMT significantly changed CBF: - decreasing L-pINS, L-ACC and L-thalamus; - increasing L-PCC, lentiform nuclei, R-vaINS, R-daINS and R-vplTHAL.
(Cerritelli et al., 2017) [29]	RCT single blinded	40 healthy adults; OTA: 20, age: 27.0 ± 5.4; OAA: 20; age: 27.0 ± 5.4;	Participants randomly attended two groups: operator tactile attention (OTA) or the operator auditory attention (OAA) group.	- 3T MRI (Philips Achieva): matrix 256 × 256, FOV = 256 mm, slice thickness = 1 mm, no gap, in-plane voxel size = 1 × 1 mm, flip angle = 12°, TR = 9.7 ms, and TE = 4 ms. - BOLD fMRI data using a gradient-echo T2∗-weighted echo-planar imaging sequence: matrix 80 × 80, voxel size 3 mm × 3 mm × 3.5 mm, SENSE 1.8, TE = 30 ms, TR = 1.8 s, and 185 volumes per run. - cardiac and respiratory data were recorded.	The findings indicated that extended periods of continuous and focused tactile touch from an operator led to a notable rise in anti-correlation between certain brain regions, such as PCC and the rINS, as well as IFG. However, these changes were distinct between OTA and OAA, and were observed only after 15 min of continuous touching.
(Gay et al., 2014) [25]	RCT single blinded	24 healthy adults and were randomly divided into 3 groups: MOB: 8, age: 21.1 ± 3.2; SMT: 6, age 20.7 ± 1.8; TT: 10, age: 22.5 ± 5.9;	- SMT group received HVLA; - MOB group received spinal mobilization; - TT group received therapeutic touch control lay prone.	- 3T MRI (Philips Achieva): sagittal orientation with XYZ dimensions of 256 × 256 × 180, FOV = 240, a slice thickness of 1 mm, no gap, voxel dimensions of 1 × 1 × 1 mm, and a TR = 8.1, TE = 3.7 ms; - pulse oximeter and respirations were recorded;	MT produced effects on FC between brain regions, including: - ACC and PCC; - aINS and pINS; - THA, SI, SII, and the PAG.
(Cella et al., 2022) [23]	Cross-over trial	40 healthy volunteers; age: 20–30;	Participants underwent two techniques (CV4 plus sCV4; ST plus sST), in a random sequence. The sham maneuvers lasted for 10 min, while the active techniques were sustained until the release of the still point, typically lasting for 6 to 8 min. EEG recordings were continuously conducted throughout the techniques and a subsequent hands-off period, totaling approximately 50 min per session.	EEG placement with the 20-channel EEG System PLUS EVOLUTION (Micromed, Italy). The EEG signal was derived from the occipital O1 and O2 electrodes: electrode impedance < 10 kΩ, signals were band-filtered 0.01–100 Hz, and notch-filtered (50 Hz). The EEG was continuously recorded during each technique.	The CV4 technique notably elevated the alpha band power in the occipital area, compared to resting state data.
(Miana et al., 2013) [24]	Pilot study	10 healthy adults, age: 28 ± 3;	Participants received the CV4, sham CV4, and no-treat control conditions in a random order, once a week.	20-channel EEG system Braintech-3000 (EMSA, Medical Instruments, Rio de Janeiro, Brazil).	There was a significant increase in the alpha band power after the administration of the CV-4 technique.

Legend: ACC (anterior cingulate cortex), aINS (anterior insular cortex), BOLD (blood-oxygen-level-dependent),CG (Control group), CV4 (compression of the fourth ventricle technique), EEG (Electroencephalography), EG (Experimental group), fMRI (functional magnetic resonance imaging),IFG (inferior frontal gyrus), LBP (low back pain), FOV (field of view), L-pINS (left posterior insula), MOB (spinal mobilization), MRI (Magnetic resonance imaging), OAA (operator auditory attention), OMT (Osteopathic Manipulative Treatment), OTA (operator tactile attention), PAG (periaqueductal grey), pCASIL (pseudo-continuous arterial spin labeling), PCC (posterior cingulate cortex), pINS (posterior insular cortex), rCBF (regional cerebral blood-flow),R-daINS (right dorsal anterior insula), rMFG (right Middle frontal gyrus), R-vaINS (right ventral anterior insula), R-vplTHAL (right ventral posterior lateral thalamus), SDs (Somatic Dysfunctions), SI (primary somatosensory cortex), SII (secondary somatosensory cortex), SMT (spinal manipulative therapy), ST (sacral technique). TT (therapeutic touch control). * Age is expressed as mean ± standard deviation. ** More details are reported in Appendix A.

## Data Availability

Data are available on demand from the corresponding author.

## Appendix A

**Table A1:** Description of the main MRI parameters.

MRI Parameters	Description
Time of echo (TE)	TE (measured in milliseconds) refers to the time between applying a radiofrequency excitation pulse and the peak of the signal induced in the coil.
Repetition time (TR)	TR (measured in milliseconds) is the time from applying an excitation pulse to the application of the next pulse.
Field of view (FOV)	FOV refers to the body region scanned during MRI. It is generally expressed in units of centimeters or millimeters.
Matrix	A matrix is an array of numbers in rows and columns and it is used in MRI to determine the scan resolution.
Voxel	Voxel is the basic unit of MR reconstruction and it represents a cube of brain tissue in a 3D image.
Flip angle	The flip angle or tip angle consists of the amount of rotation the net magnetization experiences during the application of a radiofrequency pulse. It can be measured in degrees or radians.
